# The chromatin accessibility landscape of mouse oocytes during configuration transition

**DOI:** 10.1111/cpr.13733

**Published:** 2024-09-08

**Authors:** Shuai Zhu, Jiashuo Li, Xiuwan Wang, Yifei Jin, Hengjie Wang, Huiqing An, Hongzheng Sun, Longsen Han, Bin Shen, Qiang Wang

**Affiliations:** ^1^ State Key Laboratory of Reproductive Medicine and Offspring Health, Changzhou Maternity and Child Health Care Hospital, Changzhou Medical Center Nanjing Medical University Nanjing China; ^2^ Center for Global Health, School of Public Health Nanjing Medical University Nanjing China

## Abstract

The transition of chromatin configuration in mammalian oocytes from a non‐surrounded nucleolus (NSN) to a surrounded nucleolus (SN) is critical for acquiring the developmental competence. However, the genomic and epigenomic features underlying this process remain poorly understood. In the present study, we first establish the chromatin accessibility landscape of mouse oocytes from NSN to SN stage. Through the integrative analysis of multi‐omics, we find that the establishment of DNA methylation in oocytes is independent of the dynamics of chromatin accessibility. In contrast, histone H3K4me3 status is closely associated with the dynamics of accessible regions during configuration transition. Furthermore, by focusing on the actively transcribed genes in NSN and SN oocytes, we discover that chromatin accessibility coupled with histone methylation (H3K4me3 and H3K27me3) participates in the transcriptional control during phase transition. In sum, our data provide a comprehensive resource for probing configuration transition in oocytes, and offer insights into the mechanisms determining chromatin dynamics and oocyte quality.

## Graphical abstract

This study elucidated the chromatin accessibility landscape across the non‐surrounded nucleolus (NSN) to surrounded nucleolus (SN) transition in oocyte configuration. It highlights the autonomy of DNA methylation from chromatin accessibility, contrasting with the dependency of H3K4me3. The interplay between chromatin accessibility and histone modification in transcription regulation during oocyte development is also explored.

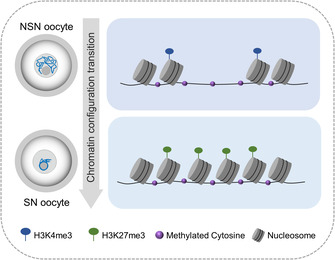

## 1 INTRODUCTION

The production of high‐quality mature oocytes constitutes the foundation for female fertility.^1^, ^2^, ^3^ In mammals, oogenesis, which encompasses the process of oocyte formation, development and maturation, initiates during foetal development and culminates years later at the time of ovulation.^4^ In the post‐natal ovary, fully‐grown oocytes are arrested at the diplotene stage of the first meiotic prophase, also known as the germinal vesicle (GV) stage. Stimulated by a surge in gonadotropins, immature oocytes resume meiosis, marked by germinal vesicle breakdown. Along with chromatin condensation and microtubule reorganisation, the oocytes advance through the first meiotic division (MI) and subsequently arrest at metaphase II (MII) until fertilisation. The intricately coordinated sequence of oogenesis is crucial for yielding developmentally competent eggs.^5^

During the late stages of oogenesis, the chromatin enclosed within the GV is subjected to several levels of regulation controlling both its structure and function, as evidenced by chromatin configuration transition from non‐surrounded nucleolus (NSN) to surrounded nucleolus (SN).^6^, ^7^, ^8^, ^9^ The NSN and SN configurations are distinguished by their chromatin organisation; the former features diffuse chromatin with sparse, dot‐like dense regions in the nucleus, whereas the latter exhibits condensed chromatin forming a ring around the nucleolus.^10^ In fact, the different chromatin configurations observed through a fluorescence microscope also possess functional significance in oocyte developmental competence and transcriptional activity. While both NSN and SN oocytes can resume meiosis and achieve maturation, NSN oocytes typically lead to developmental arrest at the two‐cell embryo stage, contrasting with SN oocytes, which are capable of developing to full term.^11^, ^12^ Furthermore, changes in oocyte chromatin configuration influence transcription and are temporally linked to the onset of transcriptional silencing.^13^, ^14^, ^15^, ^16^ Hence, the successful transition from NSN to SN is crucial for oocyte development.

The alteration in chromatin configuration correlates with a wide range of molecular behaviours and functions. It has been implicated that oocytes, during configuration transition, undergo extensive alterations in epigenetic signatures, such as histone modification and DNA methylation.^17^, ^18^, ^19^ Immunostaining analysis showed that SN oocytes exhibit the increased histone H3K4me3 methylation compared to NSN oocytes.^20^, ^21^ DNA methylation levels, indicated by 5‐methylcytosine signals, are also higher in SN than that in NSN oocytes.^20^ Furthermore, histone modifications associated with both active and repressive chromatin appear to accompany the large‐scale chromatin remodelling observed during the NSN–SN transition.^22^ These epigenetic changes may function in the regulation of transcriptional activity. However, the links between morphological changes in chromatin configuration and the epigenomic features remain poorly understood.

In this study, we systematically characterised the dynamics of chromatin state from NSN to SN stage using multi‐omics joint profiling. In particular, the correlation between chromatin accessibility and DNA/histone methylation was evaluated. Moreover, we presented the evidence showing that open chromatin coupled with elevated histone methylation participates in the transcriptional control during the configuration transition.

## 2 MATERIALS AND METHODS

### 2.1 Mice

Three‐week‐old female C57BL/6J mice were acquired from the Animal Core Facility of Nanjing Medical University. All experimental procedures involving animals were conducted in compliance with ethical guidelines and received approval from the Animal Care and Use Committee of Nanjing Medical University (Animal Use Permit Number: SYXK 2020‐0022; IACUC Approval Number: 2206014).

### 2.2 Oocyte classification and culture

Cumulus‐oocyte‐complexes were retrieved from the ovaries by manual rupturing of antral follicles, and the surrounded cumulus cells were removed by repeated mouth pipetting. For classifying chromatin configuration in GV oocytes, they were stained for 15 min with 0.1 μg/mL Hoechst 33342 in M2 medium containing 2.5 μM milrinone, subsequently sorted by exposing them to Ultraviolet light for approximately 1 s using a fluorescence microscope. The chromatin exhibited diffuse Hoechst staining interspersed with dense punctate areas, classified as NSN configuration. Chromatin condensation around the nucleolus, forming a ring‐like structure, characterises the SN configuration. Oocytes that defied categorisation were excluded from subsequent experiments. For in vitro maturation, sorted oocytes were cultured in M16 medium (Nanjing Luanchuang Co., China) under mineral oil at 37°C in a 5% CO_2_ incubator.

### 2.3 5‐Ethynyl uridine labelling and staining

To assess transcriptional activity, oocytes were cultured in M16 medium supplemented with 1 mM 5‐ethynyl uridine (EU) and 2.5 μM milrinone at 37°C for 45 min. Subsequently, the oocytes were fixed in 3.7% paraformaldehyde for 15 min, followed by permeabilization with 0.5% Triton X‐100 for 15 min. The oocytes were then incubated in the Click‐iT cocktail (C10329; Invitrogen) at room temperature for 30 min, followed by nuclear staining with Hoechst 33342. The samples were observed using a confocal laser scanning microscope (LSM 700, Zeiss), and the mean EU signal across the center of each oocyte was quantified using Image‐J.

### 2.4 In vitro fertilisation and embryo culture

In vitro fertilisation (IVF) assays were performed following the methodology outlined in our previous work. Briefly, sperm were collected from the dissected epididymides of 15‐week‐old C57BL/6 mice, and were allowed to capacitate for 1 h in toyoda‐yokoyama‐hosi medium (Nanjing Luanchuang Co., China). After a 4‐h incubation with mature oocytes for fertilisation in human tubal fluid medium, the resultant zygotes were cultured in kalium simplex optimized medium (Nanjing Luanchuang Co., China) at 37°C within a humidified atmosphere comprising 5% CO_2_, 5% O_2_, and 90% N_2_.

### 2.5 ATAC‐seq library preparation

Assay of transposase accessible chromatin (ATAC)‐seq libraries of GV oocytes were prepared in accordance with the method previously described by Zhang et al.,^23^ albeit with minor modifications. Initially, oocytes were cultured in M2 medium containing 2.5 μM milrinone and 5 μg/mL cytochalasin B for 15 min. The GV nucleus was precisely extracted with minimal cytoplasmic carryover utilising a Piezo impact‐driven micromanipulator. After washing with 0.5% PVA/PBS, 50 nuclei were added to 50 μL of lysis buffer (100 mM Tris–HCl, 10 mM NaCl, 3 mM MgCl_2_, 0.5% NP‐40 and 0.05% digitonin) for 10 min, and then subjected to tagmentation in trueprep tagment buffer L mix (TD711, Vazyme) for 0.5 h at 37°C. Five microlitres of stop buffer was added to the reaction tube and incubated for 5 min at room temperature. DNA fragments were purified using ATAC DNA extract beads, underwent library amplification for an 18‐cycle Polymerase Chain Reaction (PCR), and subsequently, the libraries were purified again using 1.2× VAHTS DNA Clean Beads.

### 2.6 DNA methylome profiling

Genome‐wide base‐resolution mapping of DNA methylation in GV oocytes using bisulfite sequencing (BS‐seq). The construction of BS‐seq libraries was adapted from the previously outlined method,^24^ with some adjustments. Twenty oocytes were collected in 2.5 μL of Buffer RLT Plus (Qiagen, 1053393) for cell lysis. The lysate was then incubated with 65 μL of Cycle Threshold (CT) conversion reagent (Zymo, d5455) for 8 min at 98°C, followed by 180 min at 65°C. The BS‐converted DNA, purified with Agencourt AMPure XP beads, received preamp oligo and 50 U of Klenow exo‐polymerase was added for preamplification, which was then subjected to exonuclease I treatment and subsequent preamplification purification. Double‐tagged products were generated through PCR with adapter oligo. Finally, the libraries were constructed by a 15‐cycle of PCR amplification using Illumina universal indexed primers.

### 2.7 CUT&Tag library generation

CUT&Tag for H3K4me3 and H3K27me3 were performed following the protocol as previously described,^25^ with minor adaptations. Briefly, 200 oocytes freed of zona pellucida were incubated with concanavalin‐coated magnetic beads (Vazyme, TD904) for 15 min at room temperature. This was followed by incubation with 1 μg of either H3K4me3 (Diagenode, C15410003) or H3K27me3 (Diagenode, C15410195) antibody at 4°C overnight, and subsequently with goat anti‐rabbit IgG antibody (Vazyme, Ab207) for 1 h. The samples were incubated with 0.08 μM pA/G‐Tnp Pro on a rotator at room temperature for 1 h, subsequently resuspended in 50 μL of Trueprep Tagment Buffer L, and incubated at 37°C for 1 h. After tagmentation, DNA extraction was performed using DNA Extract Beads Pro. Finally, the libraries were constructed through 17 cycles of PCR amplification using the TruePrep Index Kit for Illumina (Vazyme, TD202).

### 2.8 RNA‐seq

The transcriptome analysis of GV oocytes was conducted via Smart‐seq2, following our previously described methodology.^26^ Fifteen oocytes were lysed in 0.2% Triton X‐100 containing 80 U RNase Inhibitor. First‐strand cDNA synthesis involved the Oligo(dT) (G, A, or C) and (A, T, G, or C) primer, followed by the 5′TS oligo primer. Subsequently, cDNA amplification was carried out using an ISPCR primer in an 18‐cycle PCR, and the products underwent purification via VAHTS DNA Clean Beads (Vazyme, N411). Quality control for the amplification products involved measuring cDNA concentration with Qubit 4 and assessing cDNA fragment distribution with Qsep 400. Following this, sequencing libraries were prepared with the TruePrep DNA Library Prep Kit for Illumina (Vazyme, TD503).

### 2.9 Quantitative real‐time PCR

The mRNA levels of indicated genes were assessed through quantitative real‐time PCR (RT‐PCR) with the comparative CT method, as we previously described.^27^ Total RNAs were extracted from 30 oocytes employing RNeasy® Micro Kit (Invitrogen, 157030297) and cDNA synthesis was completed using HiScript III real‐time SuperMix for qPCR (Vazyme, R323). RT‐PCR was performed with SYBR Green (Vazyme, Q111) in a final reaction volume of 20 μL using an ABI QuantStudio™ 7 Flex PCR system (Applied Biosystems). Relative mRNA levels were normalised to the level of *Gapdh* mRNA (internal control). The related primers sequences are listed in Table S4.

### 2.10 Data processing

#### 2.10.1 DNA methylation analysis

The raw reads were trimmed by trim_galore (V0.6.10) with default parameters to remove adaptors and low‐quality bases. Trimmed reads were aligned by using Bismark^28^ (V0.24.1) against the mouse reference genome GRCm39 with parameters: ‐‐non_directional ‐‐bowtie2 ‐‐bam ‐‐single_end. PCR duplicates were filtered by Deduplicate_bismark with default parameters. Only high‐quality reads (Mapping Qualities >10) were retained for further analysis.

The DNA methylation level of individual cytosine sites within the reference genome sequence was determined as the percentage of reads supporting the presence of a methylated cytosine (C). Unless specified, the DNA methylation level of CpG sites within a given sample or region reflects the overall DNA methylation level of that particular sample or region. Given the use of low‐input cells, analysis of DNA methylation levels was conducted based on 1X read coverage. Additionally, the DNA methylation level was analysed by using CGmapTools^29^ (V0.1.3).

To identify differentially methylated regions (DMRs), metilene^30^ (V 0.2‐8) was utilised with default parameters, and genome annotation was carried out using ChIPseeker^31^ (V 1.38.0) with parameters: tssRegion = *c*(−20,000) downstream distance = 0.

#### 2.10.2 RNA‐seq analysis

Adaptors and low‐quality bases were removed by using trim_galore (v0.6.10) with default parameters from raw reads. Retained reads were mapped to mouse reference genome (GRCm39) by using HISAT2^32^ (v2.2.0) with gencode annotation (vM34). Read quantification was carried out with FeatureCounts^33^ (v2.0.6) (parameters: ‐t exon ‐g gene_id ‐Q 10 ‐‐primary ‐s 0 ‐p ‐‐countReadPairs) and normalised to fragments per kilobase of transcript per million mapped values using customised script. To determine differential expression genes, DESeq2^34^ (V1.40.1) was used with parameters: foldchange ≥1.2, padj <0.05.

#### 2.10.3 CUT&Tag and ATAC‐seq data analysis

Raw reads were trimmed using trim_galore (v0.6.10) and mapped to the mouse reference genome (GRcm39) using Bowtie2^35^ (V2.5.1) with parameters: ‐X 2000 ‐I 10. All unmapped reads, non‐uniquely mapped reads, and PCR duplicates were removed. The signal tracks and heatmap were both generated by deepTools^36^ (V3.5.2). Given the low‐input cells and the nature of oocytes, the histone modification data between two stages were randomly subsampled to an equal number of reads and the broad peaks were called using GoPeaks^37^ (V1.0.0) with parameters: ‐‐broad ‐‐mdist 3000. For ATAC‐seq, Genrich (V0.6.1, https://github.com/jsh58/Genrich) was used to identify open genomic regions with parameters: ‐j ‐D. Then genome annotation was carried out using ChIPseeker^31^ (V1.38.0) with parameters: tssRegion = *c*(−20,000) downstream distance = 0.

#### 2.10.4 Gene annotation

During data analysis, we used GENCODE GRCm39 gene annotation vM34.

#### 2.10.5 Identification of dynamic genomic regions in chromatin accessibility

Given the low‐input cells and the validity of the data, the two sets of ATAC‐seq peaks identified in NSN and SN stages were used to identify dynamic genomic regions (Open‐Close regions, Open‐Open regions and Close‐Open regions) using bedtools^38^ (V2.31.1). Then genome annotation was carried out using ChIPseeker^31^ (V1.38.0) with parameters: tssRegion = *c*(−20,000) downstream distance = 0.

#### 2.10.6 Motif enrichment analysis

All dynamic regions that are at least 2 kb away from annotated transcription start site (TSSs) were selected as distal regions; otherwise, they were clustered as proximal regions. And then motif enrichment analysis was performed on distal and proximal regions by HOMER^39^ (V 4.11).

#### 2.10.7 Identification of actively transcribed genes

Based on our KAS‐seq data,^40^ the single‐strand DNA (ssDNA) normalised read density (Reads Per Kilobase per Million mapped reads) of genes bodies was calculated by deepTools at each stage, and the differential genes were determined by DESeq2. Finally, the differential genes bound by RNA polymerase II (Pol II) were considered as actively transcribed genes (ATGs).

### 2.11 Statistical analysis

The data are presented as the mean ± standard deviation unless otherwise specified. Student's *t*‐test was used for comparisons of quantitative characteristics between two groups. Data analysis was conducted using GraphPad Prism 7.0 software. The significance level for all tests was set at *p* < 0.05.

## 3 RESULTS

### 3.1 Impact of chromatin configuration on transcriptional activity and developmental competence in oocytes

**FIGURE 1:**
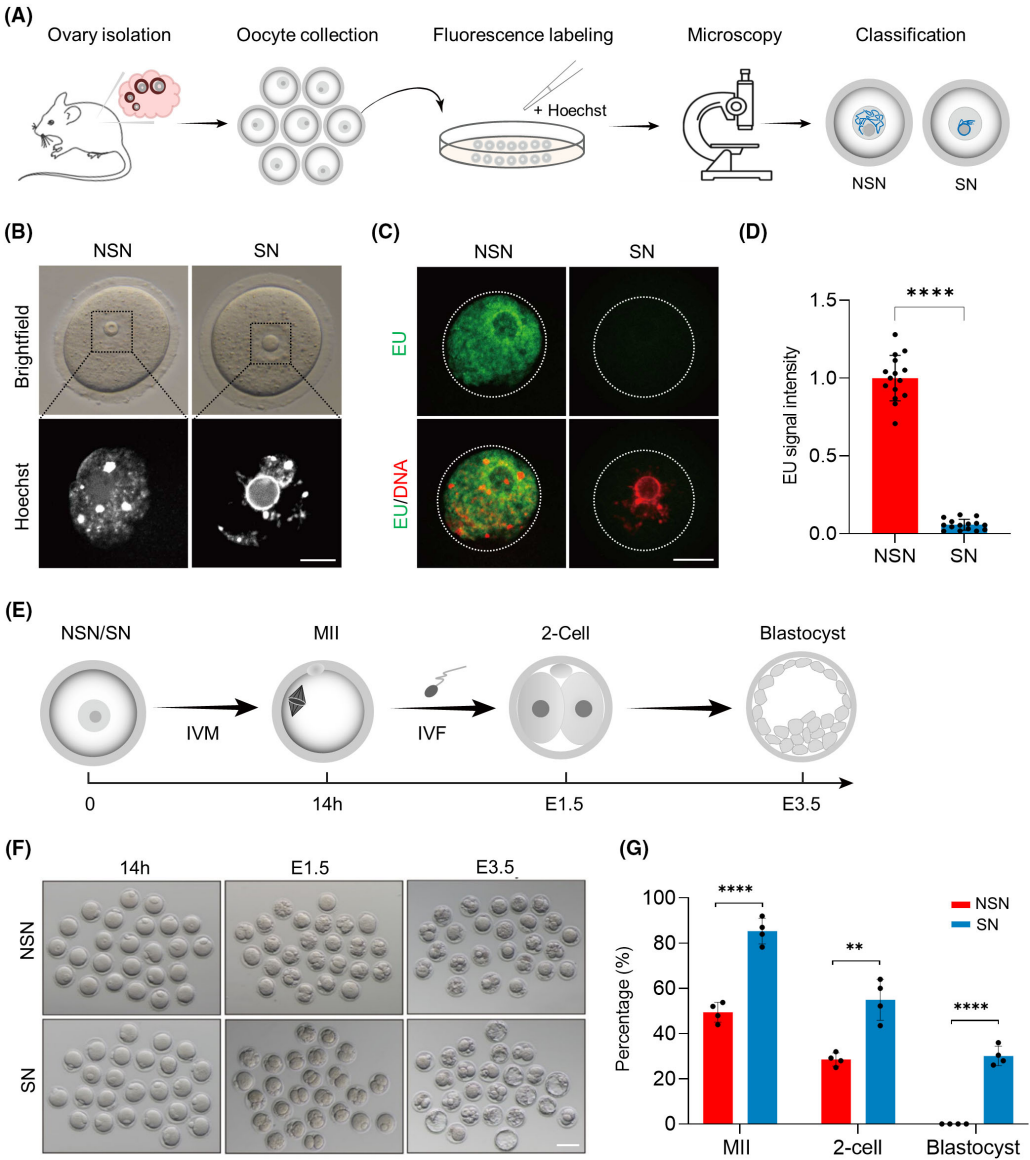
Impact of chromatin configuration on transcriptional activity and developmental competence in oocyte. (A) Schematic of the workflow for classification of chromatin configuration in mouse oocytes. (B) Representative images of the non‐surrounded nucleolus (NSN) and surrounded nucleolus (SN) oocytes stained with Hoechst 33342. Scale bar, 20 μm. (C) Representative images of 5‐ethynyl uridine (EU) staining in oocytes. The white dashed circles represent the nucleus of oocytes. Scale bar, 10 μm. (D) Quantification of EU signal intensity in oocytes. Each dot represents a single oocyte. EU signals were normalised to NSN oocytes. (E) Schematic diagram of in vitro maturation (IVM) and in vitro fertilisation (IVF) experiments. (F) Representative bright‐field images of NSN and SN‐derived oocytes and E1.5 and E3.5 embryos. Scale bar, 80 μm. (G) The percentage of NSN and SN‐derived oocytes and embryos that successfully progressed to the MII, two‐cell and blastocyst stage at 14 h, E1.5 and E3.5 during in vitro culture. Data are presented as means ± SD from four independent experiments. Throughout, a student's *t*‐test (two‐tailed) was used for statistical analysis. ****p < 0.0001; **p < 0.01.

GV oocytes were collected from the antral follicles and freed of cumulus cells. Then they were stained with the Hoechst 33342 and examined under a fluorescence microscope for individual classification into NSN and SN types, based on the degree and spatial arrangement of chromatin (Figure 1A). Specifically, NSN oocytes feature chromatin dispersed throughout the nucleus, whereas SN oocytes display highly condensed chromatin forming a ring‐like structure around the nucleolus (Figure 1B). Using EU labelling to visualise newly synthesised RNA, we observed that SN oocytes present nearly undetectable EU signals (Figure 1C,D), indicating a dramatic decrease in transcriptional activity during chromatin remodelling. To evaluate the impact of chromatin configuration on developmental competence, oocytes were cultured for in vitro maturation and fertilisation (Figure 1E). We found that both NSN and SN oocytes could achieve meiotic maturation; however, the rate of polar body 1 (Pb1) extrusion was significantly lower in NSN oocytes (85.3% vs. 49.4%, Figure 1F,G), indicative of the disrupted maturational progression. Moreover, 30.1% of zygotes derived from SN oocytes could develop into blastocyst; in contrast, most zygotes derived from NSN oocytes experienced the developmental arrest at the two‐cell embryo stage (Figure 1F,G). Together, these results underscore the importance of chromatin configuration in determining oocyte transcriptional activity and developmental competence.

### 3.2 Global features of chromatin accessibility in oocytes during configuration transition

**FIGURE 2:**
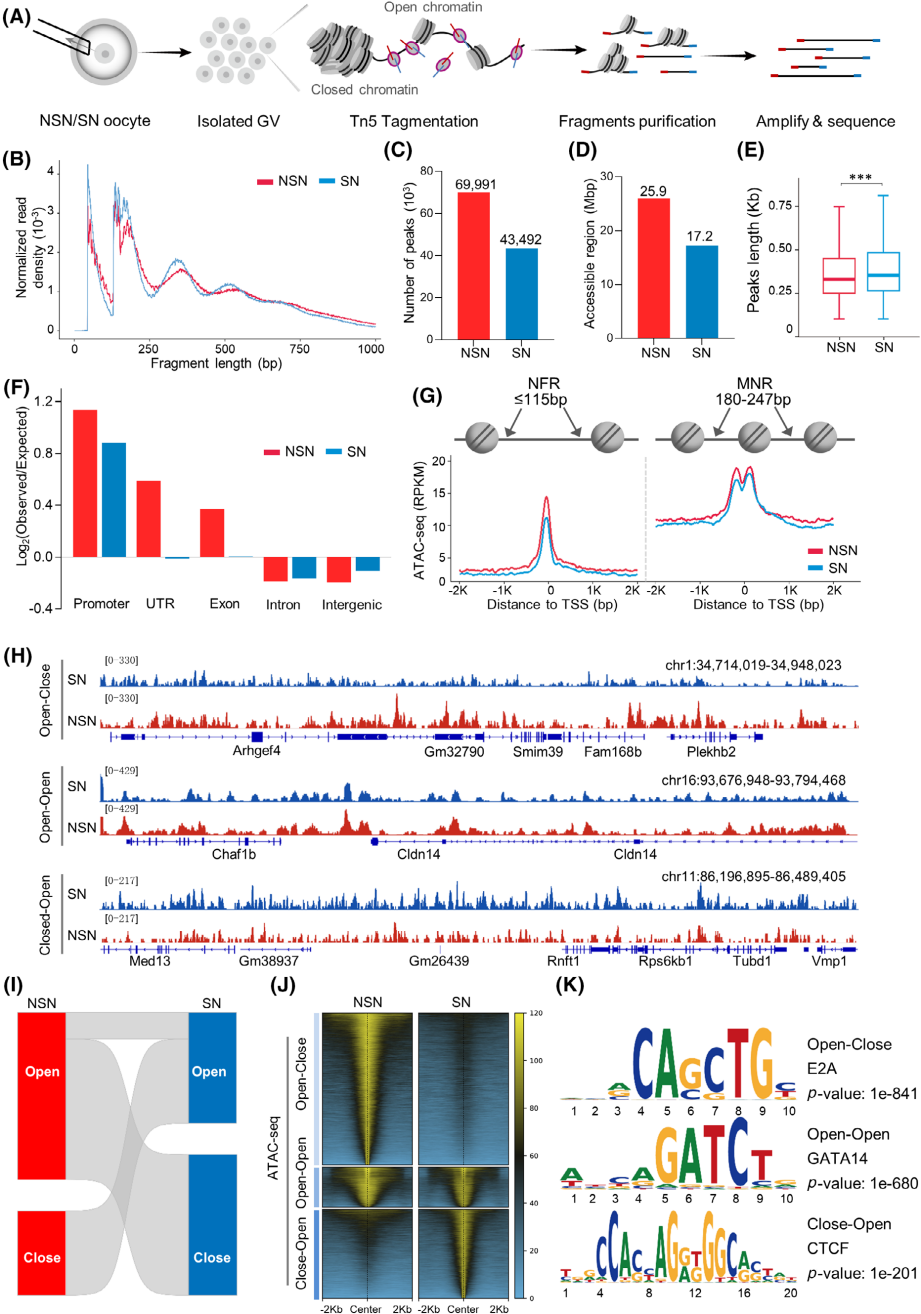
Global features of chromatin accessibility in oocytes during configuration transition. (A) Schematic of ATAC‐seq for probing accessible chromatin in mouse GV oocytes. (B) ATAC‐seq fragment size distribution corresponding to non‐surrounded nucleolus (NSN) and surrounded nucleolus (SN). (C) The number of peaks detected in indicated oocytes. (D) Analysis of accessible region sizes. (E) Box plots illustrating the average peak lengths. *p*‐Values based on the two‐sided Wilcoxon test. ***p < 0.001. (F) Genomic distribution of peaks for NSN and SN oocytes. (G) Plots of the average ATAC‐seq enrichment around the TSS of genes profiled by mono‐nucleosome regions (MNR) and nucleosome‐free regions (NFR). (H) Genome browser view of ATAC‐seq in the representative Open‐Close, Close‐Open and Open‐Open region. (I) Sankey diagram showing the dynamic changes in chromatin accessibility between NSN and SN oocytes. (J) Heatmap of ATAC‐seq in dynamic accessible regions. (K) Significantly enriched transcription factor in distal (>2 kb from transcription start sites [TSSs]) of three dynamic accessible regions.

To fully characterise the accessible chromatin landscape during configuration transition, we conducted ATAC‐seq profiling on GVs isolated from NSN and SN oocytes, respectively (Figure 2A). The replicates display a strong and significant correlation within identical configurations (Figure S1A,B). The size distribution of ATAC‐seq fragment demonstrated clear periodicity (Figure 2B), indicative of nucleosome occupancy. We found a significant reduction in chromatin‐accessible regions transitioning from NSN to SN stage (Figure 2C–E). Notably, despite the relatively low number of peaks in SN oocyte (with 43,492 peaks and 17.2 Mbp region detected in both replicates; Figure 2C,D), the average peak length is higher than that in NSN oocytes (Figure 2E). In addition, while the genomic distribution patterns of accessible regions in NSN and SN oocytes are broadly similar (Figure S1C), SN are characterised by increased accessibility in intron and intergenic regions and decreased accessibility in untranslated region and exon regions compared to NSN (Figure 2F). In contrast, chromatin accessibility between the TSS and transcription end site (TES) diminished in SN oocytes (Figure S1D). It has been reported that transcription factors canonically bind nucleosome‐free DNA, making the positioning of nucleosomes within regulatory regions crucial to the regulation of gene expression.^41^ In order to explore the detailed characteristics of chromatin accessibility around TSS, ATAC‐seq reads within the ranges of 180–247 and ≤115 bp were utilized to distinguish nucleosome‐free regions (NFRs) and mono‐nucleosome regions (MNRs).^42^ Importantly, we found the reduced enrichment of NFRs around the TSS in both NSN and SN oocytes compared to MNRs. Furthermore, both NFRs and MNRs were enriched around TSSs in NSN oocytes relative to SN oocytes (Figure 2G), indicative of the active regulation of transcription in NSN oocytes. The ATAC‐seq data not only show the global differences in chromatin accessibility between NSN and SN oocytes, but also identify the specific alterations in diverse genomic regions.

Next, we analysed the dynamics of chromatin accessibility during configuration transition. All accessible regions identified in NSN and SN oocytes (termed dynamic accessible regions in this paper) were categorised into three types: Open‐Close (open in NSN but closed in SN), Close‐Open (closed in NSN but open in SN) and Open‐Open (open in both NSN and SN) (Figure 2H and Table S1). Of them, the Open‐Close type has the highest proportion, accounting for approximately 50.7% of the total accessible regions, whereas the Close‐Open type comprises 27.1%, and the Open‐Open type constitutes only 22.2% (Figure 2I,J). The comparative analysis of the genomic distribution of these dynamic regions revealed that the Open‐Close is more prevalent in promoter regions relative to other two types (Figure S2A). Close‐Open has a higher proportion in intron regions, and Open‐Open is predominantly found in intergenic areas (Figure S2A). Moreover, by employing the motif analysis software HOMER,^39^ we investigated the enrichment of potential transcription factors within dynamic accessible regions, categorised into proximal (<2 kb from TSSs) and distal (>2 kb from TSSs) based on their proximity to TSSs. A significant enrichment of CCCTC‐binding factor (CTCF) in both the proximal and distal of Closed‐Open chromatin regions was discovered (Figures 2K and S2B). Altogether, these data imply that chromatin accessibility in oocytes undergoes dynamic alterations during configuration transition.

### 3.3 Establishment of genomic methylation in oocytes is independent of the dynamics of chromatin accessibility

**FIGURE 3:**
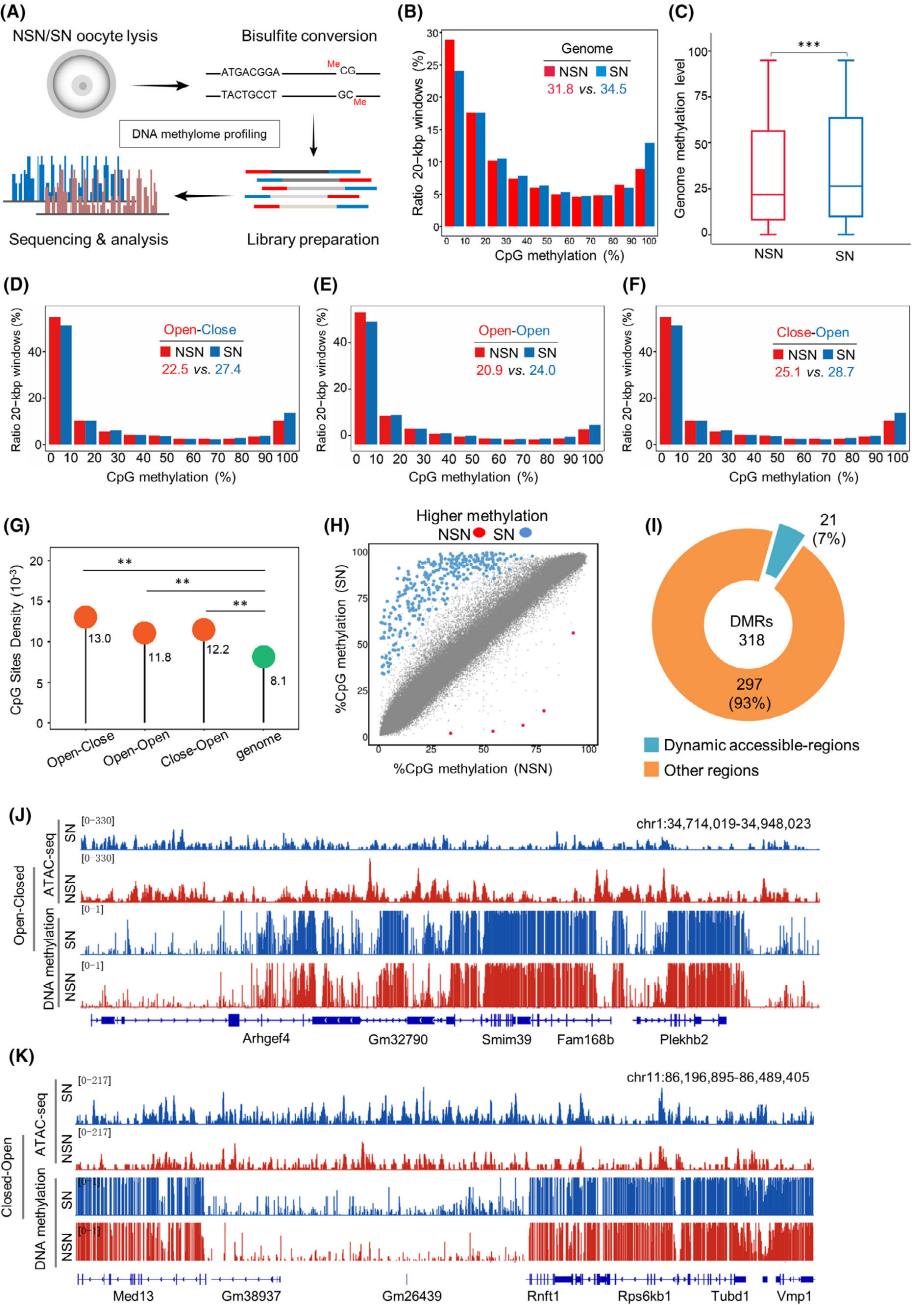
Establishment of genomic methylation in oocytes is independent of the dynamics of chromatin accessibility. (A) Flow chart illustrating the bisulfite sequencing procedure for genome‐wide methylation analysis. Non‐surrounded nucleolus (NSN) and surrounded nucleolus (SN) oocytes were collected, DNA was bisulfite‐converted, and libraries were prepared and subjected to high‐throughput sequencing. (B) Distribution of 20‐kbp genomic windows in NSN and SN oocytes according to their percentage of DNA methylation. Mean methylation levels are marked by the numerical value. (C) Boxplot of average CpG methylation levels in NSN and SN oocytes. *p*‐Values were calculated using a two‐sided Wilcoxon test. ***p < 0.001. (D–F) The pattern of DNA methylation distribution in three dynamic accessible regions at NSN and SN stage. (G) Lollipop plot showing the mean CpG sites density levels of three dynamic accessible regions and the whole genome, indicated by numerical values. *t*‐Test (two‐tailed) was used for statistical analysis. **p < 0.01. (H) Scatter plot showing the average methylation of differentially methylated regions (DMRs) and non‐DMRs. SN hyper‐DMRs, 313; NSN hyper‐DMRs, 5. nCpGs ≥10, *q* value ≤0.05. (I) The proportion of DMRs in dynamic accessible regions. (J, K) The Integrative Genomics Viewer browser view showing the ATAC‐seq enrichment and CpG methylation levels in Close‐Open and Open‐Close regions, respectively.

Throughout the process of oocyte growth and development, the landscape of DNA methylation undergoes profound remodelling.^43^ To evaluate the potential coordination between chromatin accessibility and DNA methylation in NSN and SN oocytes, BS‐seq analysis was carried out (Figure 3A). Although both types of oocytes displayed a clear bimodal distribution of CpG methylation (Figure 3B), the global CpG methylation level in NSN oocytes is significantly lower than that in SN oocytes (Figure 3C; *p*< 0.001). Next, we analysed the DNA methylation features in those dynamic, accessible regions. Intriguingly, the CpG methylation levels of all three areas (Open‐Close, Close‐Open and Open‐Open) are drastically reduced in comparison to the average level of genomic DNA (Figure 3D–F). Nonetheless, the density of CpG sites is relatively higher in these areas (Figure 3G). These observations strongly indicate that there may be the existence of the specific methylation features in the dynamic accessible regions during configuration transition.

Moreover, we asked whether the changes in chromatin accessibility during configuration transition contribute to the establishment of DMRs in oocytes. To address this question, 318 high‐confidence DMRs were identified across the genome, with the majority (313, 98%) showing the elevated methylation in SN oocytes (Figure 3H). However, only 21 (7%) DMRs were located in the dynamic accessible regions (Figure 3I). Combining that all these dynamic accessible regions experienced the comparable trend of methylation changes (Figures 3J,K and S3), the chromatin accessibility seems not to significantly influence genomic methylation in oocytes.

### 3.4 Chromatin accessibility is associated with H3K4me3 during configuration transition

**FIGURE 4:**
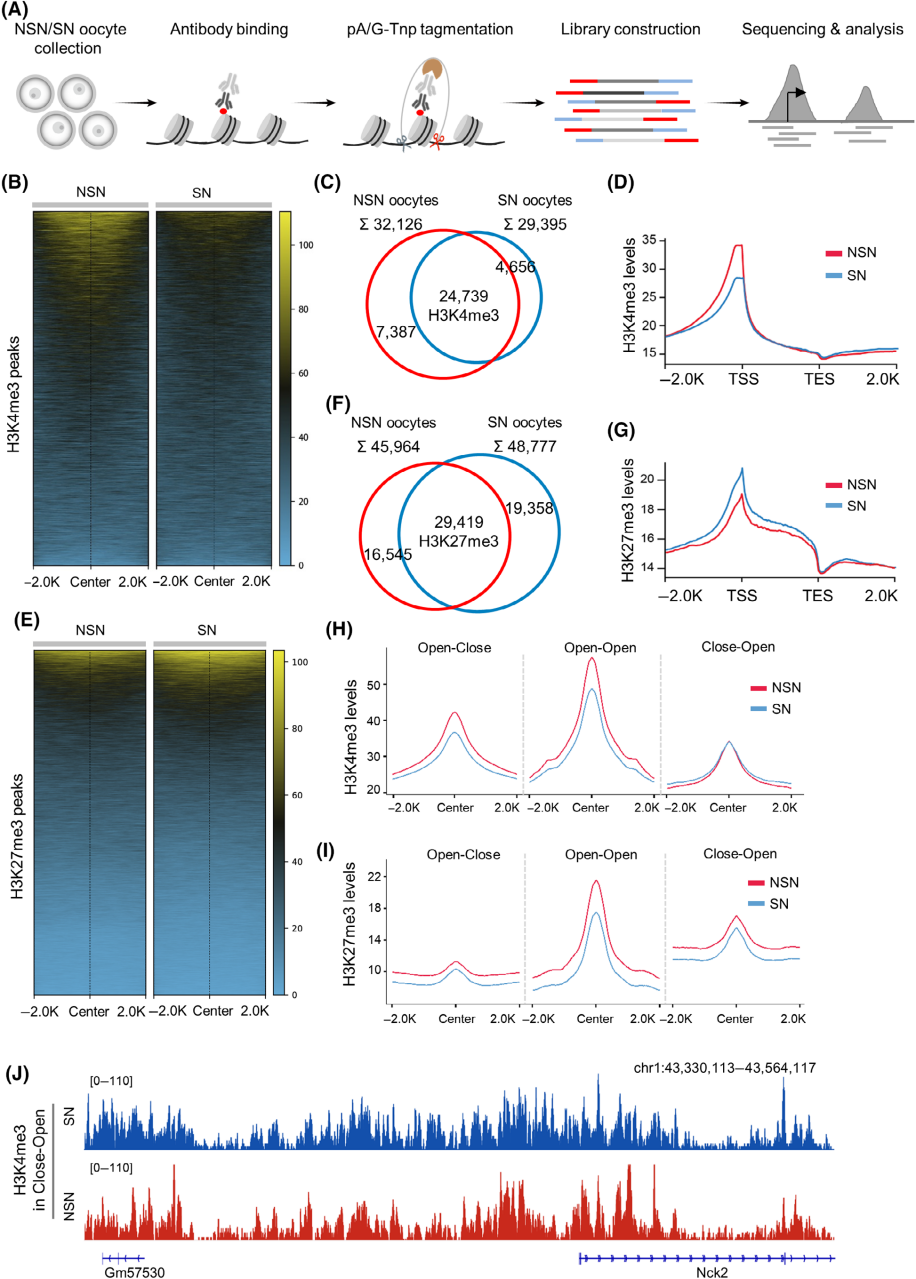
Chromatin accessibility is associated with H3K4me3 during configuration transition. (A) Schematic of CUT&Tag sequencing of H3K4me3 and H3K27me3 in NSN and SN oocytes. (B) Heatmaps showing enrichment of H3K4me3 in non‐surrounded nucleolus (NSN) and surrounded nucleolus (SN) oocytes. (C) Venn diagram showing the overlapped H3K4me3 peaks between NSN and SN. (D) The plot of H3K4me3 enrichment at transcription start site (TSS) and transcription end site (TES). Transcripts scaled to 2 kb, 2 kb upstream and downstream regions shown. (E) Heatmaps showing enrichment of H3K27me3 in NSN and SN oocytes. (F). Venn diagram showing the overlapped H3K27me3 peaks between NSN and SN. (G) The plot of H3K27me3 enrichment at TSS and TES. Transcripts scaled to 2 kb, 2 kb upstream and downstream regions shown. (H) The enrichment of H3K4me3 in dynamic accessible regions. (I) The enrichment of H3K27me3 in dynamic accessible regions. (J) The Integrative Genomics Viewer browser view showing the H3K4me3 levels in Close‐Open regions.

A myriad of histone post‐translational modifications (PTMs) integrate signalling information into chromatin to regulate access to and expression of DNA.^44^, ^45^ This interplay indicates the crucial correlation between histone PTMs and the modulation of chromatin accessibility. The presence of bivalent modifications, represented by the co‐occurrence of methylated histones associated with gene activation (histone H3 lysine 4 trimethylation, H3K4me3) and repression (histone H3 lysine 27 trimethylation, H3K27me3), is hypothesised to predispose genes for either expression or silencing.^46^ Here, through the CUT&Tag sequencing, we mapped the genome‐wide profiles of H3K4me3 and H3K27me3 in NSN and SN oocytes, respectively (Figures 4A and S4A,B). As shown in Figure 4B, we noted that the enrichment of H3K4me3 was evidently higher in NSN than that in SN, and about 67% of the peaks were overlapped between them (Figure 4C). In particular, H3K4me3 was significantly enriched around the TSSs in NSN oocytes compared to SN oocytes (Figure 4D). In striking contrast, H3K27me3 was found to be more enriched in SN oocytes relative to NSN oocytes (Figure 4E), and 45% of peaks were overlapped (Figure 4F). Different from H3K4me3, H3K27me3 enrichment around the TSSs was detected in SN oocytes (Figure 4G).

To dissect the potential relationship between histone methylation and chromatin conformation in oocytes, the profiling characteristics of H3K4me3 and H3K27me3 in the dynamic accessible regions were examined. We discovered that H3K4me3 levels are reduced in Open‐Close regions where chromatin accessibility is lost from NSN to SN (Figures 4H and S5A). Meanwhile, H3K4me3 exhibited the highest levels in those maintained open regions (Open‐Open; Figure 4H), and the minimum for the regions that become accessible in SN (Close‐Open Figure 4H,J). However, we did not observe the corresponding changes in H3K27me3 levels that were consistent with the dynamics of accessible regions and instead just showing a declining trend (Figures 4I and S5B). Collectively, these findings indicate that chromatin accessibility is closely associated with H3K4me3 in oocytes.

### 3.5 Chromatin status participates in the transcriptional control during configuration transition

Although transcriptional silence from the NSN to SN stage has been implicated by EU staining^47^ (Figure 1C), the molecular correlation between chromatin accessibility and transcriptional activity remains to be explored. To this end, we performed RNA‐seq to evaluate the transcript abundance between NSN and SN oocytes (Figure S6A), and a total of 1437 differentially expressed genes were identified (Figure S6B and Table S2). Accuracy of the transcriptome data was validated by qRT‐PCR of mRNA levels for randomly selected genes (Figure S6C). Interestingly, we found that almost 46% of them are upregulated in SN oocytes, indicative of transcript accumulation. This observation suggests that transcriptome data are unable to accurately reflect the transcriptional activity in oocytes.

**FIGURE 5:**
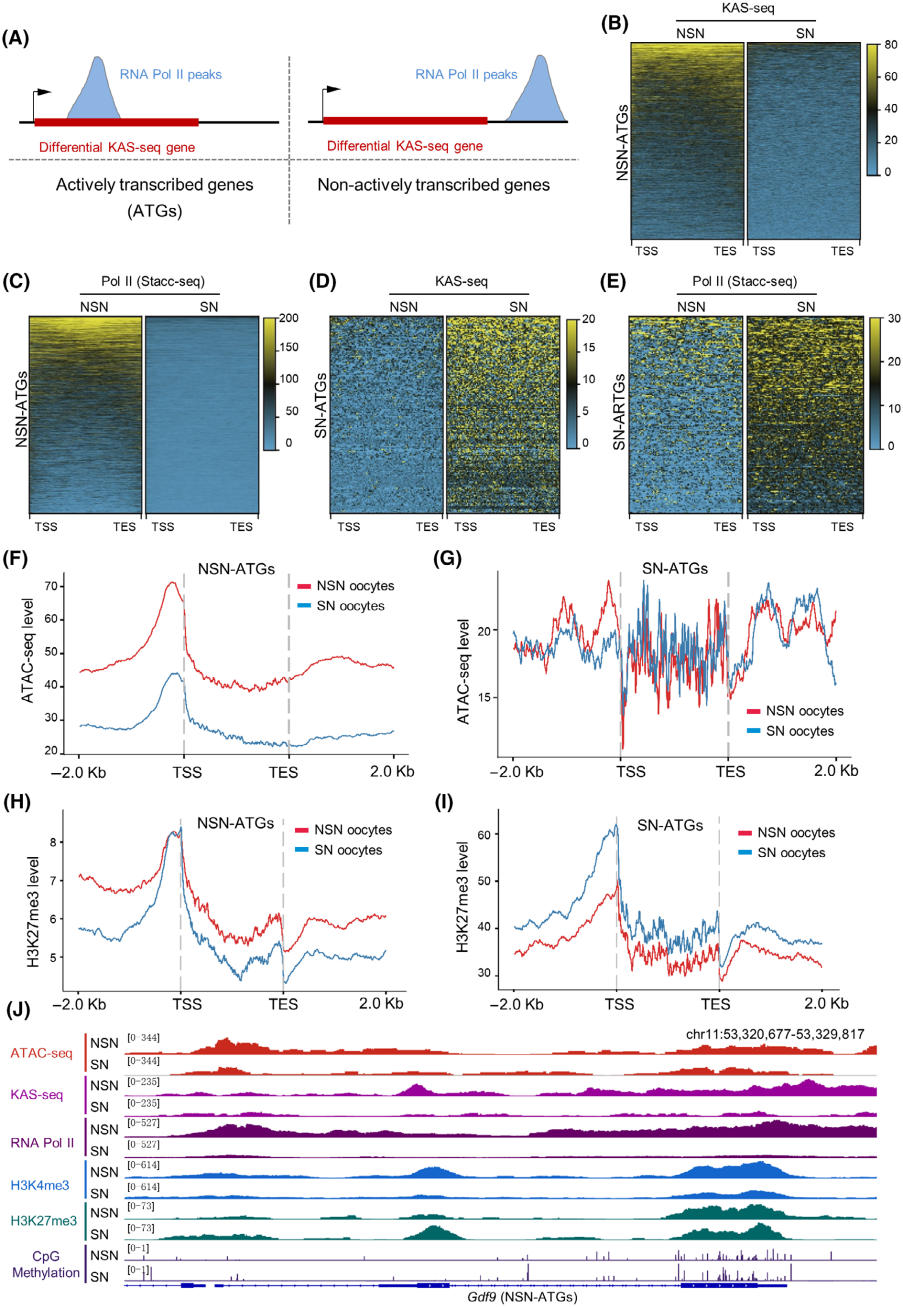
Chromatin status participates in the transcriptional control during the configuration transition. (A) Schematic illustration of identifying the actively transcribed genes (non‐surrounded nucleolus [NSN]‐actively transcribed genes [ATGs] and surrounded nucleolus [SN]‐ATGs). Genes with high KAS‐seq read density and RNA polymerase II (Pol II) binding on gene bodies are considered ATGs. (B) Heatmaps showing the distribution of KAS‐seq signals for NSN‐ATGs. (C) Heatmaps displaying the enrichment of RNA Pol II (Stacc‐seq) for NSN‐ATGs. (D) Heatmaps showing the distribution of KAS‐seq signals for SN‐ATGs. (E) Heatmaps displaying the enrichment of RNA Pol II (Stacc‐seq) for SN‐ATGs. (F) The enrichment level of ATAC‐seq for NSN‐ATGs. (G) The enrichment level of ATAC‐seq for SN‐ATGs. (H) The enrichment level of H3K27me3 for NSN‐ATGs. (I) The enrichment level of H3K27me3 for SN‐ATGs. Loci scaled to 2 kb, regions 2 kb upstream and downstream shown. (J) The Integrative Genomics Viewer browser view showing the ATAC‐seq, KAS‐seq, RNA Pol II, H3K4me3, H3K27me3 and CpG methylation enrichment near *Gdf9* (NSN‐ATGs). TES, transcription end site; TSS, transcription start site.

In eukaryotic cells, RNA Pol II is involved in the initiation and elongation of gene transcription.^48^, ^49^ Small‐scale Tn5‐assisted chromatin cleavage with sequencing (Stacc‐seq) has been used to investigate the landscapes of Pol II binding in mouse embryos.^50^ In addition, KAS‐seq, a technique that enables rapid and sensitive labelling of ssDNA produced in transcription regions via a kethoxal‐guanine reaction for sequencing, enhances the understanding of global transcriptional activity.^51^ Here, by integrative analysis of Pol II data published by Liu et al.^50^ and our KAS‐seq data,^40^ we identified 2575 ATGs in this paper (Figure 5A and Table S3) in fully‐grown immature oocytes. Notably, 92.7% (2388) of them displayed the active transcription in NSN oocytes (termed NSN‐ATGs; Figure 5B,C), and 7.3% (187; SN‐ATGs) are active in SN oocytes (Figure 5D,E). Next, we decided to determine whether the chromatin state during the configuration transition affects gene transcription in oocytes. As shown in Figure 5F, we found that the chromatin accessibility in the gene body of NSN‐ATGs is markedly reduced from NSN to SN stage (i.e., *Gdf9* in Figure 5J). In contrast, the chromatin accessibility of those SN‐ATGs (Figure 5G) was slightly elevated from NSN to SN stage (i.e., *Lhcgr* in Figure S7A). The observations indicate that chromatin open may promote the transcription activity during configuration transition in oocytes. In addition, similar to chromatin accessibility, both NSN‐ATGs and SN‐ATGs presented the significant enrichment of H3K27me3 (Figure 5H,I) and H3K4me3 (Figure S7B,C). However, the DNA methylation levels of all ATGs did not show any association with their transcriptional activity (Figure S7D,E). Taking together, these findings suggest that chromatin accessibility coupled with histone methylation participates in the transcriptional control during configuration transition.

## 4 DISCUSSION

**FIGURE 6:**
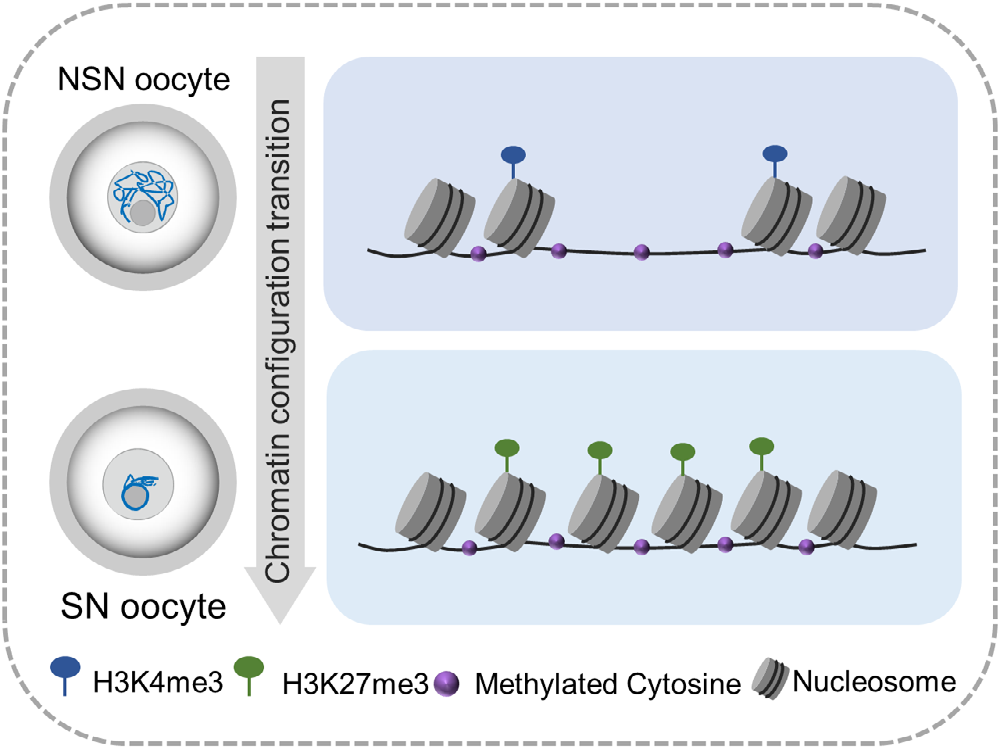
A diagram indicates the underlying epigenomic features of oocytes during configuration transition. NSN, non‐surrounded nucleolus; SN, surrounded nucleolus.

During the late stages of oogenesis, GV oocytes experience chromatin remodelling marked by transcriptional silencing, transitioning from NSN to SN configuration. This transition is critical for the acquisition of meiotic maturation and developmental competence in oocytes.^52^ In this study, we correlated the morphological aspects of chromatin condensation with the underlying genomic and epigenomic features (Figure 6). The ATAC‐seq analysis unveiled a global reduction in chromatin accessibility during configuration transition (Figure 2). Significantly, our analysis revealed that approximately 27% of the total accessible chromatin regions observed was newly established in SN oocytes, underscoring the intricate nature of chromatin dynamics. Furthermore, we discovered an enrichment of CTCF‐binding motifs within regions transitioning from a closed to an open state (Figure 2), suggesting a pivotal role in chromatin restructuring. Previous studies have demonstrated that the oocyte‐specific depletion of the catalytic subunits of CTCF‐binding partners SWI/SNF complex, SMARCA4^53^ and SMARCA5,^23^ results in female subfertility or infertility, manifesting as defects in oocyte meiosis and subsequent embryonic development arrest. Given the critical function of CTCF in organising chromatin architecture and regulating gene transcription,^54^ further studies are warranted to unravel the precise mechanisms through which CTCF influences the chromatin configuration transition in mouse oocytes.

Physical access to DNA is a highly dynamic property of chromatin regulated by epigenetics, such as DNA methylation and histone modifications.^55^ We analysed the DNA methylome, as well as the H3K4me3 and H3K27me3 in the dynamic accessible regions (Figures 3 and 4). Throughout the transition from NSN to SN, we observed a general elevation in DNA methylation levels across the genome. Intriguingly, dynamic accessible regions exhibited relatively lower methylation levels compared to whole genome, indicating the unique methylation characteristics in these areas. Nonetheless, the developmental significance of methylation changes in dynamic accessible regions needs to be uncovered in the future. In mammals, the regulation of H3K4me3 is highly precise and governs the processes of oocyte growth and development.^56^, ^57^, ^58^ H3K4me3 is abundantly deposited at numerous broad regions on the chromatin of mature oocytes,^59^ yet its connection with chromatin accessibility in oocytes remains unclear. Our data clearly showed the correlations between the chromatin accessibility and H3K4me3 modification, particularly notable in Close‐Open region (Figure 4). Exploring the enzymes responsible for H3K4me3 methylation will be helpful for understanding the regulatory mechanism of chromatin accessibility during chromatin remodelling.

Alterations in gene transcription activity accompanying chromatin configuration transition were implicated through EU labelling.^16^, ^60^ Transcriptome analysis, which primarily targets mRNAs with poly(A) tails, does not fully capture the real‐time transcriptional status of genes. Substantial amounts of mRNA accumulate in oogenesis to facilitate maturation and early embryonic development.^61^ Thus, our findings indicate a minimal correlation between the expression of differentially expressed genes identified via RNA‐seq and the extent of chromatin accessibility in oocytes (Figure S6). Of note, our integrative analysis of ATAC‐seq, RNA pol II stacc‐seq and KAS‐seq discovered numerous genes that display real‐time transcriptional activity in NSN and SN oocytes (Figure 5). Gene Ontology analysis of NSN‐ATGs highlighted the clustering of genes related to chromatin remodelling (GO:0006338) and epigenetic regulation of gene expression (GO:0040029), while SN‐ATGs were mainly enriched in cellular metabolism terms, such as inositol phosphate metabolic process (GO:0043647) and polyol metabolic process (GO:0019751). Furthermore, the dynamic changes in accessibility indeed influence the transcription of these genes during configuration transition in oocytes. Two important questions need to be addressed in the future: (i) to establish the molecular networks connecting chromatin accessibility, histone modifications and transcriptional control in oocytes; (ii) to investigate the effects of dynamic changes in oocyte chromatin accessibility on the subsequent oocyte maturation and embryo development.

In summary, our study established a landscape of chromatin accessibility in mouse oocytes during configuration transition, and revealed its potential correlation with histone methylation and transcriptional activity through the multi‐omics analysis. This work sheds light on the molecular events associated with oocyte developmental potential and paves the way for the identification of molecular markers indicative of oocyte quality.

## AUTHOR CONTRIBUTIONS

SZ, JL and QW conceived and designed the project. BS performed the ATAC‐seq library construction and sequencing. SZ performed BS‐seq, RNA‐seq and CUT&Tag library construction and sequencing. JL and XW analysed the data. SZ, YJ, HW, HA, HS and LH performed the mouse oocyte experiments. SZ and JL wrote and QW revised the manuscript. All authors reviewed and approved the manuscript for publication.

## FUNDING INFORMATION

This work was supported by the National Natural Science Foundation of China (No. 81925014 and 82221005 to QW; No. 82401945 to SZ), the National Key Research and Development Program of China (No. 2021YFC2700400 to QW), and the Natural Science Foundation of Jiangsu Province (BK20230058 to LH).

## CONFLICT OF INTEREST STATEMENT

The authors have declared no competing interests.

## Supporting information


**Data S1.**Supporting Information.

Table S1.

Table S2.

Table S3.

Table S4.

## Data Availability

The datasets used and/or analysed during the current study are available from the corresponding author (QW) on reasonable request. The accession numbers of the ATAC‐seq, BS‐seq, CUT&Tag and RNA‐seq data generated in this work are GEO: GSE261972 (password: ureboycahrkpzsv), GSE262346 (bqbscyyprepxix), GSE262263 (mxwtyisajvctvmb) and GSE262265 (gjmvyyugnfizjiz). RNA Pol II Stacc‐seq data set was GSE135457 from Liu et al.^50^

## References

[1] Keefe D , Kumar M , Kalmbach K . Oocyte competency is the key to embryo potential. Fertil Steril. 2015;103(2):317‐322. 25639967 10.1016/j.fertnstert.2014.12.115

[2] Innocenti F , Fiorentino G , Cimadomo D , et al. Maternal effect factors that contribute to oocytes developmental competence: an update. J Assist Reprod Genet. 2022;39(4):861‐871. 35165782 10.1007/s10815-022-02434-y PMC9051001

[3] Gosden RG . Oogenesis as a foundation for embryogenesis. Mol Cell Endocrinol. 2002;186(2):149‐153. 11900888 10.1016/s0303-7207(01)00683-9

[4] Wang Q , Sun QY . Evaluation of oocyte quality: morphological, cellular and molecular predictors. Reprod Fertil Dev. 2007;19(1):1‐12. 10.1071/rd06103 17389130

[5] Conti M , Franciosi F . Acquisition of oocyte competence to develop as an embryo: integrated nuclear and cytoplasmic events. Hum Reprod Update. 2018;24(3):245‐266. 29432538 10.1093/humupd/dmx040 PMC5907346

[6] Zuccotti M , Piccinelli A , Giorgi Rossi P , Garagna S , Redi CA . Chromatin organization during mouse oocyte growth. Mol Reprod Dev. 1995;41(4):479‐485. 7576615 10.1002/mrd.1080410410

[7] Monti M , Calligaro A , Behr B , Rejo Pera R , Redi CA , Wossidlo M . Functional topography of the fully grown human oocyte. Eur J Histochem. 2017;61(1):2769. 28348419 10.4081/ejh.2017.2769 PMC5304266

[8] Sun XS , Liu Y , Yue KZ , Ma SF , Tan JH . Changes in germinal vesicle (GV) chromatin configurations during growth and maturation of porcine oocytes. Mol Reprod Dev. 2004;69(2):228‐234. 15293225 10.1002/mrd.20123

[9] Tan JH , Wang HL , Sun XS , Liu Y , Sui HS , Zhang J . Chromatin configurations in the germinal vesicle of mammalian oocytes. Mol Hum Reprod. 2009;15(1):1‐9. 19019837 10.1093/molehr/gan069

[10] Mattson BA , Albertini DF . Oogenesis: chromatin and microtubule dynamics during meiotic prophase. Mol Reprod Dev. 1990;25(4):374‐383. 1691651 10.1002/mrd.1080250411

[11] Zuccotti M , Giorgi Rossi P , Martinez A , Garagna S , Forabosco A , Redi CA . Meiotic and developmental competence of mouse antral oocytes. Biol Reprod. 1998;58(3):700‐704. 9510956 10.1095/biolreprod58.3.700

[12] Inoue A , Nakajima R , Nagata M , Aoki F . Contribution of the oocyte nucleus and cytoplasm to the determination of meiotic and developmental competence in mice. Hum Reprod. 2008;23(6):1377‐1384. 18367455 10.1093/humrep/den096

[13] Bouniol‐Baly C , Hamraoui L , Guibert J , Beaujean N , Szollosi MS , Debey P . Differential transcriptional activity associated with chromatin configuration in fully grown mouse germinal vesicle oocytes. Biol Reprod. 1999;60(3):580‐587. 10026102 10.1095/biolreprod60.3.580

[14] Ma JY , Li M , Luo YB , et al. Maternal factors required for oocyte developmental competence in mice: transcriptome analysis of non‐surrounded nucleolus (NSN) and surrounded nucleolus (SN) oocytes. Cell Cycle. 2013;12(12):1928‐1938. 23673344 10.4161/cc.24991 PMC3735707

[15] Sui X , Hu Y , Ren C , et al. METTL3‐mediated m^6^A is required for murine oocyte maturation and maternal‐to‐zygotic transition. Cell Cycle. 2020;19(4):391‐404. 31916488 10.1080/15384101.2019.1711324 PMC7100890

[16] Xia M , He H , Wang Y , et al. PCBP1 is required for maintenance of the transcriptionally silent state in fully grown mouse oocytes. Cell Cycle. 2012;11(15):2833‐2842. 22801551 10.4161/cc.21169

[17] Sasaki H , Matsui Y . Epigenetic events in mammalian germ‐cell development: reprogramming and beyond. Nat Rev Genet. 2008;9(2):129‐140. 18197165 10.1038/nrg2295

[18] Eleftheriou K , Peter A , Fedorenko I , Schmidt K , Wossidlo M , Arand J . A transition phase in late mouse oogenesis impacts DNA methylation of the early embryo. Commun Biol. 2022;5(1):1047. 36184676 10.1038/s42003-022-04008-1 PMC9527251

[19] Gu C , Liu S , Wu Q , Zhang L , Guo F . Integrative single‐cell analysis of transcriptome, DNA methylome and chromatin accessibility in mouse oocytes. Cell Res. 2019;29(2):110‐123. 30560925 10.1038/s41422-018-0125-4 PMC6355938

[20] Kageyama S , Liu H , Kaneko N , Ooga M , Nagata M , Aoki F . Alterations in epigenetic modifications during oocyte growth in mice. Reproduction. 2007;133(1):85‐94. 17244735 10.1530/REP-06-0025

[21] Zuccotti M , Bellone M , Longo F , Redi CA , Garagna S . Fully‐mature antral mouse oocytes are transcriptionally silent but their heterochromatin maintains a transcriptional permissive histone acetylation profile. J Assist Reprod Genet. 2011;28(12):1193‐1196. 21468653 10.1007/s10815-011-9562-4 PMC3241842

[22] Bonnet‐Garnier A , Feuerstein P , Chebrout M , et al. Genome organization and epigenetic marks in mouse germinal vesicle oocytes. Int J Dev Biol. 2012;56(10–12):877‐887. 23417410 10.1387/ijdb.120149ab

[23] Zhang C , Chen Z , Yin Q , et al. The chromatin remodeler Snf2h is essential for oocyte meiotic cell cycle progression. Genes Dev. 2020;34(3–4):166‐178. 31919188 10.1101/gad.331157.119 PMC7000916

[24] Clark SJ , Smallwood SA , Lee HJ , Krueger F , Reik W , Kelsey G . Genome‐wide base‐resolution mapping of DNA methylation in single cells using single‐cell bisulfite sequencing (scBS‐seq). Nat Protoc. 2017;12(3):534‐547. 28182018 10.1038/nprot.2016.187

[25] Kaya‐Okur HS , Wu SJ , Codomo CA , et al. CUT&tag for efficient epigenomic profiling of small samples and single cells. Nat Commun. 2019;10(1):1930. 31036827 10.1038/s41467-019-09982-5 PMC6488672

[26] Han L , Chen Y , Li L , et al. Increased mtDNA mutation frequency in oocytes causes epigenetic alterations and embryonic defects. Natl Sci Rev. 2022;9(10):nwac136. 36325113 10.1093/nsr/nwac136 PMC9616472

[27] Hou X , Zhu S , Zhang H , et al. Mitofusin1 in oocyte is essential for female fertility. Redox Biol. 2019;21:101110. 30690319 10.1016/j.redox.2019.101110 PMC6351231

[28] Krueger F , Andrews SR . Bismark: a flexible aligner and methylation caller for bisulfite‐Seq applications. Bioinformatics. 2011;27(11):1571‐1572. 21493656 10.1093/bioinformatics/btr167 PMC3102221

[29] Guo W , Zhu P , Pellegrini M , Zhang MQ , Wang X , Ni Z . CGmapTools improves the precision of heterozygous SNV calls and supports allele‐specific methylation detection and visualization in bisulfite‐sequencing data. Bioinformatics. 2018;34(3):381‐387. 28968643 10.1093/bioinformatics/btx595 PMC6454434

[30] Jühling F , Kretzmer H , Bernhart SH , Otto C , Stadler PF , Hoffmann S . Metilene: fast and sensitive calling of differentially methylated regions from bisulfite sequencing data. Genome Res. 2016;26(2):256‐262. 26631489 10.1101/gr.196394.115 PMC4728377

[31] Yu G , Wang LG , He QY . ChIPseeker: an R/bioconductor package for ChIP peak annotation, comparison and visualization. Bioinformatics. 2015;31(14):2382‐2383. 25765347 10.1093/bioinformatics/btv145

[32] Zhang Y , Park C , Bennett C , Thornton M , Kim D . Rapid and accurate alignment of nucleotide conversion sequencing reads with HISAT‐3N. Genome Res. 2021;31(7):1290‐1295. 34103331 10.1101/gr.275193.120 PMC8256862

[33] Liao Y , Smyth GK , Shi W . featureCounts: an efficient general purpose program for assigning sequence reads to genomic features. Bioinformatics. 2014;30(7):923‐930. 24227677 10.1093/bioinformatics/btt656

[34] Love MI , Huber W , Anders S . Moderated estimation of fold change and dispersion for RNA‐seq data with DESeq2. Genome Biol. 2014;15(12):550. 25516281 10.1186/s13059-014-0550-8 PMC4302049

[35] Langmead B , Salzberg SL . Fast gapped‐read alignment with bowtie 2. Nat Methods. 2012;9(4):357‐359. 22388286 10.1038/nmeth.1923 PMC3322381

[36] Ramírez F , Ryan DP , Grüning B , et al. deepTools2: a next generation web server for deep‐sequencing data analysis. Nucleic Acids Res. 2016;44(W1):W160‐W165. 27079975 10.1093/nar/gkw257 PMC4987876

[37] Yashar WM , Kong G , VanCampen J , et al. GoPeaks: histone modification peak calling for CUT&tag. Genome Biol. 2022;23(1):144. 35788238 10.1186/s13059-022-02707-w PMC9252088

[38] Quinlan AR , Hall IM . BEDTools: a flexible suite of utilities for comparing genomic features. Bioinformatics. 2010;26(6):841‐842. 20110278 10.1093/bioinformatics/btq033 PMC2832824

[39] Heinz S , Benner C , Spann N , et al. Simple combinations of lineage‐determining transcription factors prime cis‐regulatory elements required for macrophage and B cell identities. Mol Cell. 2010;38(4):576‐589. 20513432 10.1016/j.molcel.2010.05.004 PMC2898526

[40] An H , Wang X , Li J , et al. KAS‐seq profiling captures transcription dynamics during oocyte maturation. J Ovarian Res. 2024;17(1):23. 38267939 10.1186/s13048-023-01342-8 PMC10807090

[41] Schep AN , Buenrostro JD , Denny SK , Schwartz K , Sherlock G , Greenleaf WJ . Structured nucleosome fingerprints enable high‐resolution mapping of chromatin architecture within regulatory regions. Genome Res. 2015;25(11):1757‐1770. 26314830 10.1101/gr.192294.115 PMC4617971

[42] Buenrostro JD , Giresi PG , Zaba LC , Chang HY , Greenleaf WJ . Transposition of native chromatin for fast and sensitive epigenomic profiling of open chromatin, DNA‐binding proteins and nucleosome position. Nat Methods. 2013;10(12):1213‐1218. 24097267 10.1038/nmeth.2688 PMC3959825

[43] Smallwood SA , Kelsey G . De novo DNA methylation: a germ cell perspective. Trends Genet. 2012;28(1):33‐42. 22019337 10.1016/j.tig.2011.09.004

[44] Millán‐Zambrano G , Burton A , Bannister AJ , Schneider R . Histone post‐translational modifications—cause and consequence of genome function. Nat Rev Genet. 2022;23(9):563‐580. 35338361 10.1038/s41576-022-00468-7

[45] Bannister AJ , Kouzarides T . Regulation of chromatin by histone modifications. Cell Res. 2011;21(3):381‐395. 21321607 10.1038/cr.2011.22 PMC3193420

[46] Macrae TA , Fothergill‐Robinson J , Ramalho‐Santos M . Regulation, functions and transmission of bivalent chromatin during mammalian development. Nat Rev Mol Cell Biol. 2023;24(1):6‐26. 36028557 10.1038/s41580-022-00518-2

[47] Zhang J , Zhang YL , Zhao LW , et al. Mammalian nucleolar protein DCAF13 is essential for ovarian follicle maintenance and oocyte growth by mediating rRNA processing. Cell Death Differ. 2018;26(7):1251‐1266. 30283081 10.1038/s41418-018-0203-7 PMC6748096

[48] Segall J , Matsui T , Roeder RG . Multiple factors are required for the accurate transcription of purified genes by RNA polymerase III. J Biol Chem. 1980;255(24):11986‐11991. 7440579

[49] Zhou Q , Li T , Price DH . RNA polymerase II elongation control. Annu Rev Biochem. 2012;81:119‐143. 22404626 10.1146/annurev-biochem-052610-095910 PMC4273853

[50] Liu B , Xu Q , Wang Q , et al. The landscape of RNA pol II binding reveals a stepwise transition during ZGA. Nature. 2020;587(7832):139‐144. 33116310 10.1038/s41586-020-2847-y

[51] Wu T , Lyu R , You Q , He C . Kethoxal‐assisted single‐stranded DNA sequencing captures global transcription dynamics and enhancer activity in situ. Nat Methods. 2020;17(5):515‐523. 32251394 10.1038/s41592-020-0797-9 PMC7205578

[52] Briley SM , Ahmed AA , Steenwinkel TE , et al. Global SUMOylation in mouse oocytes maintains oocyte identity and regulates chromatin remodeling and transcriptional silencing at the end of folliculogenesis. Development. 2023;150(17):dev201535. 37676777 10.1242/dev.201535 PMC10499029

[53] Abedini A , Landry DA , Macaulay AD , et al. SWI/SNF chromatin remodeling subunit Smarca4/BRG1 is essential for female fertility. Biol Reprod. 2023;108(2):279‐291. 36440965 10.1093/biolre/ioac209 PMC9930400

[54] Tang Z , Luo OJ , Li X , et al. CTCF‐mediated human 3D genome architecture reveals chromatin topology for transcription. Cell. 2015;163(7):1611‐1627. 26686651 10.1016/j.cell.2015.11.024 PMC4734140

[55] Klemm SL , Shipony Z , Greenleaf WJ . Chromatin accessibility and the regulatory epigenome. Nat Rev Genet. 2019;20(4):207‐220. 30675018 10.1038/s41576-018-0089-8

[56] Dahl JA , Jung I , Aanes H , et al. Broad histone H3K4me3 domains in mouse oocytes modulate maternal‐to‐zygotic transition. Nature. 2016;537(7621):548‐552. 27626377 10.1038/nature19360 PMC6283663

[57] Sha QQ , Dai XX , Jiang JC , et al. CFP1 coordinates histone H3 lysine‐4 trimethylation and meiotic cell cycle progression in mouse oocytes. Nat Commun. 2018;9(1):3477. 30154440 10.1038/s41467-018-05930-x PMC6113306

[58] Ciccone DN , Su H , Hevi S , et al. KDM1B is a histone H3K4 demethylase required to establish maternal genomic imprints. Nature. 2009;461(7262):415‐418. 19727073 10.1038/nature08315

[59] Zhang B , Zheng H , Huang B , et al. Allelic reprogramming of the histone modification H3K4me3 in early mammalian development. Nature. 2016;537(7621):553‐557. 27626382 10.1038/nature19361

[60] Monti M , Zanoni M , Calligaro A , Ko MS , Mauri P , Redi CA . Developmental arrest and mouse antral not‐surrounded nucleolus oocytes. Biol Reprod. 2013;88(1):2. 23136301 10.1095/biolreprod.112.103887 PMC4434939

[61] Yu C , Ji SY , Sha QQ , et al. BTG4 is a meiotic cell cycle‐coupled maternal‐zygotic‐transition licensing factor in oocytes. Nat Struct Mol Biol. 2016;23(5):387‐394. 27065194 10.1038/nsmb.3204

