# Supplementary material for: The chromatin accessibility landscape of mouse oocytes during configuration transition
Source: Cell Prolif. 2024 Sep 8;58(1):e13733. doi: 10.1111/cpr.13733 (PMC11693577; doi:10.1111/cpr.13733)

Supplemental figure legends

Supplementary Figure S1. Quality control of the ATAC-seq in GV oocytes. (A) The Pearson correlation between the replicates of ATAC-seq samples at NSN and SN stages. (B) Scatter plots comparing the enrichment (20-kb window for the entire genome) between ATAC-seq replicates for NSN and SN stages. The Pearson correlation of the RPKM values and P-values calculated by two-sided Wilcoxon test are shown. (C) Bar plot showing the genomic distributions of chromatin accessibility. (D) The average levels of ATAC-seq enrichment for genes in the NSN and SN oocytes.

Supplementary Figure S2. Dynamic accessible-regions at NSN and SN oocytes. (A) Bar plot showing the genomic distributions of chromatin accessibility of each region. (B) Transcription factors motifs identified from proximal (< 2kb from TSSs) ATAC-seq peaks of dynamic accessible-regions.

Supplementary Figure S3. DNA methylation features in dynamic accessible-regions. The IGV browser view showing the signals of ATAC-seq and CpG methylation in Open-Open regions of NSN and SN oocytes.

Supplementary Figure S4. Quality control of the CUT&Tag sequencing in GV oocytes. (A) The Pearson correlation between the replicates of H3K4me3 and H3K27me3 samples. (B) Principal component plots of H3K4me3 and H3K27me3 samples in NSN and SN oocytes.

Supplementary Figure S5. H3K4me3 and H3K27me3 features in dynamic accessible-regions. (A) Heatmaps showing enrichment of H3K4me3 in the Open-Close, Close-Open and Open-Open regions. (B) Heatmaps showing enrichment of H3K27me3 in the Open-Close, Close-Open and Open-Open regions.

Supplementary Figure S6. RNA-seq analysis of NSN and SN oocytes. (A) Principal component plots of RNA-seq replicates. (B) Scatter plot showing the differentially expression genes between SN and NSN stages. (C) Validation of RNA-Seq data by quantitative RT-PCR. Data are expressed as the mean ± SD from three independent experiments. **P< 0.01.

Supplementary Figure S7. Epigenetic signatures of actively transcribed genes at NSN and SN oocytes. (A) The IGV browser view showing the signals of ATAC-seq, KAS-seq, RNA Pol II, H3K4me3, H3K27me3 and CpG methylation enrichment near *Lhcgr* (SN-ATGs). The average enrichment of H3K4me3 for NSN-ATGs (B) and SN-ATGs (C). The CpG methylation levels of NSN-ATGs (D) and SN-ATGs (E).

Supplementary Figure S1

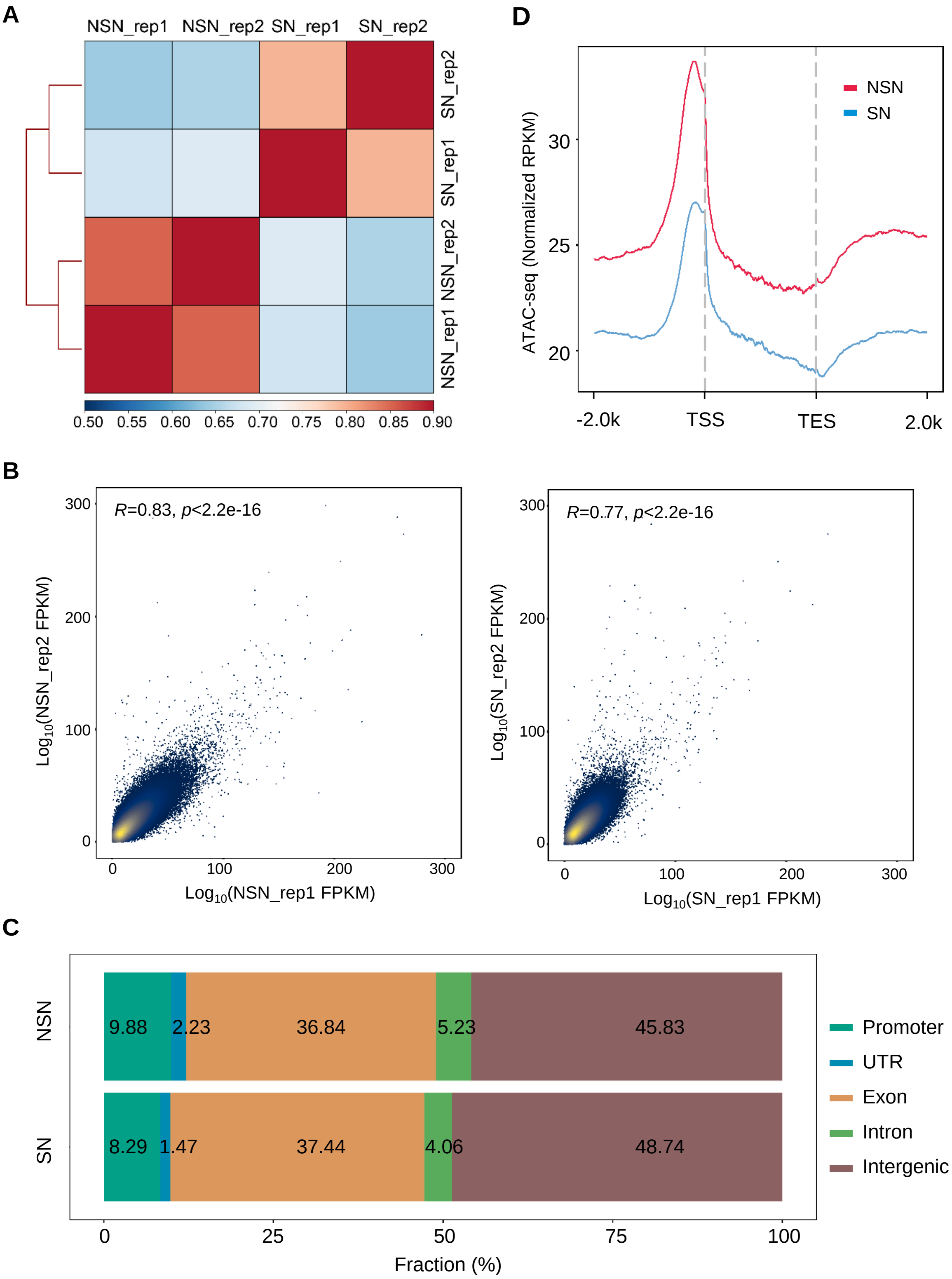

Supplementary Figure S2

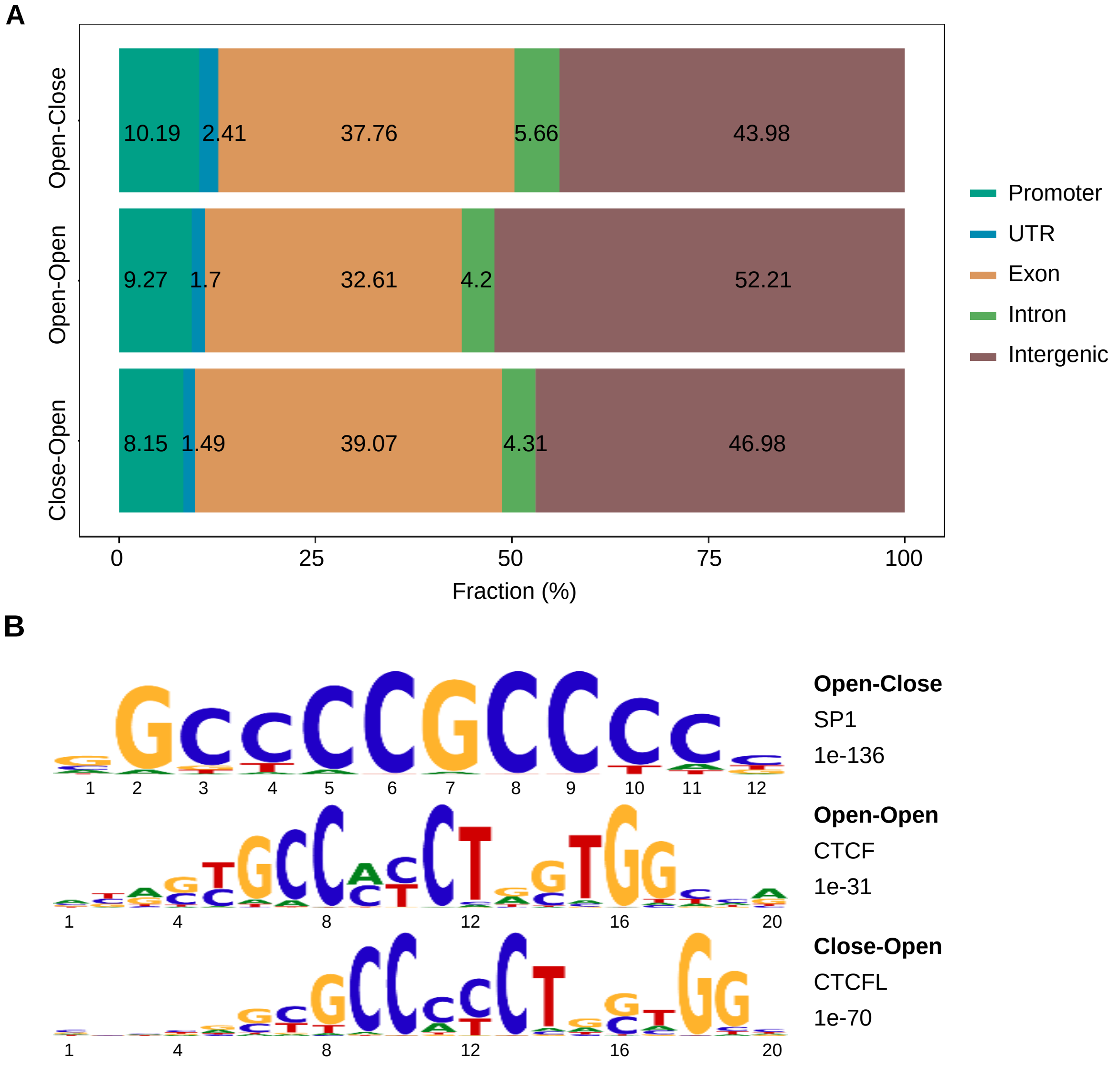

Supplementary Figure S3

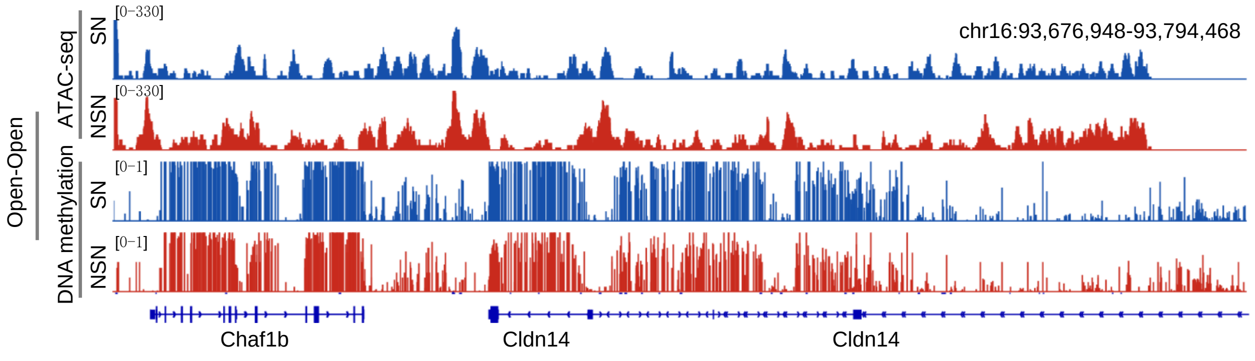

Supplementary Figure S4

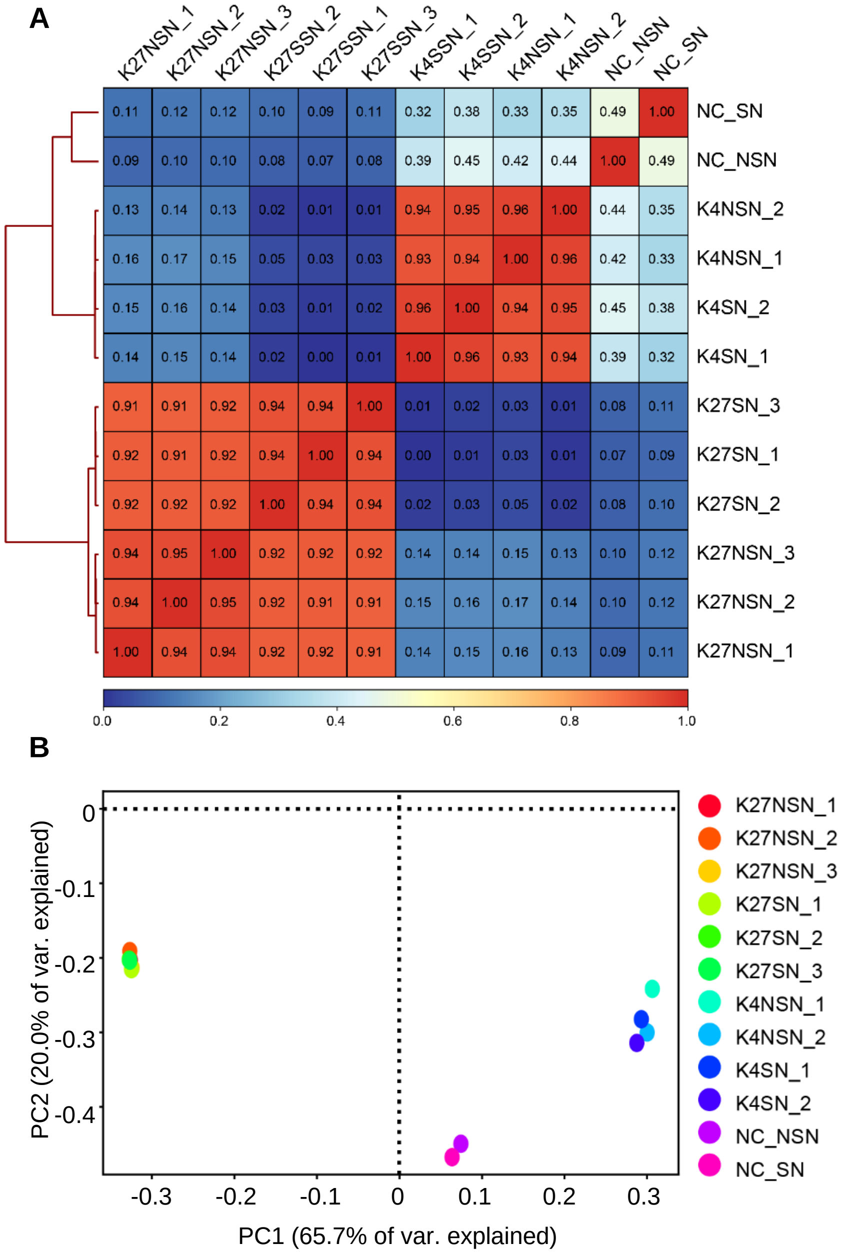

Supplementary Figure S5

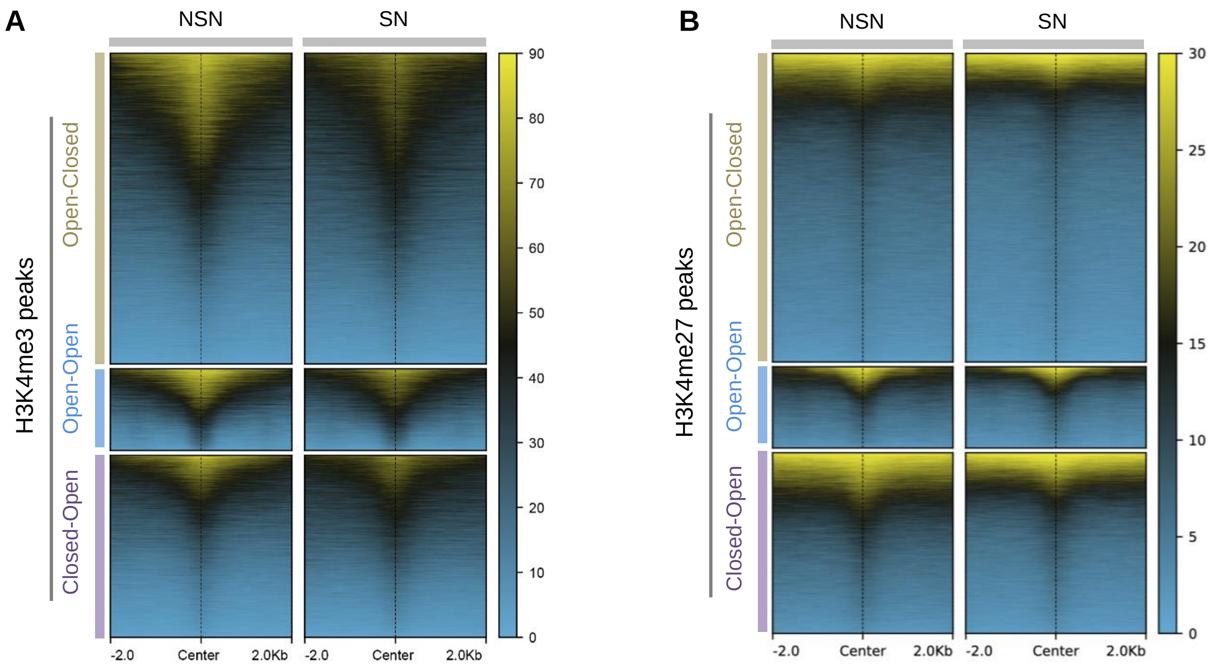

Supplementary Figure S6

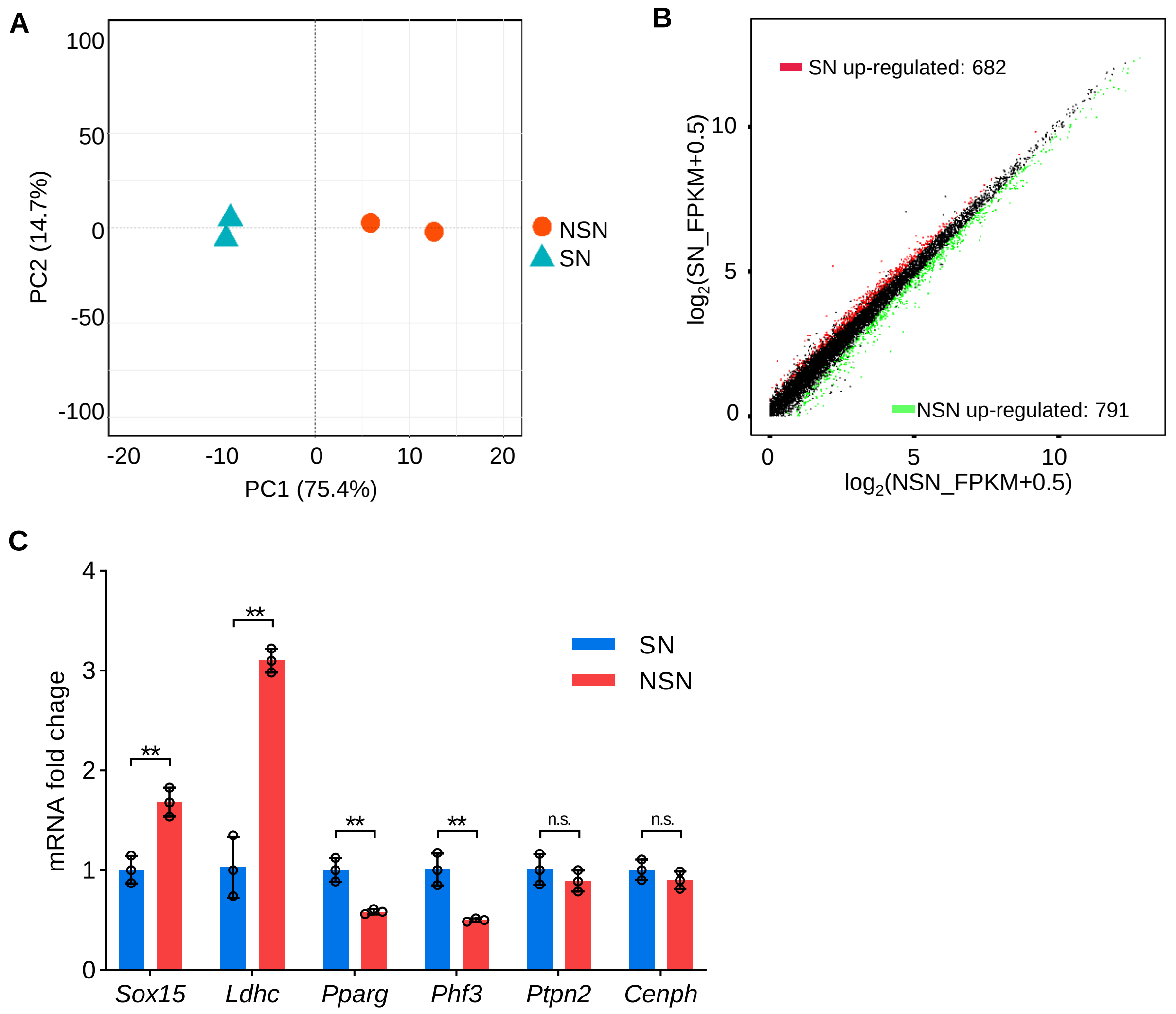

Supplementary Figure S7

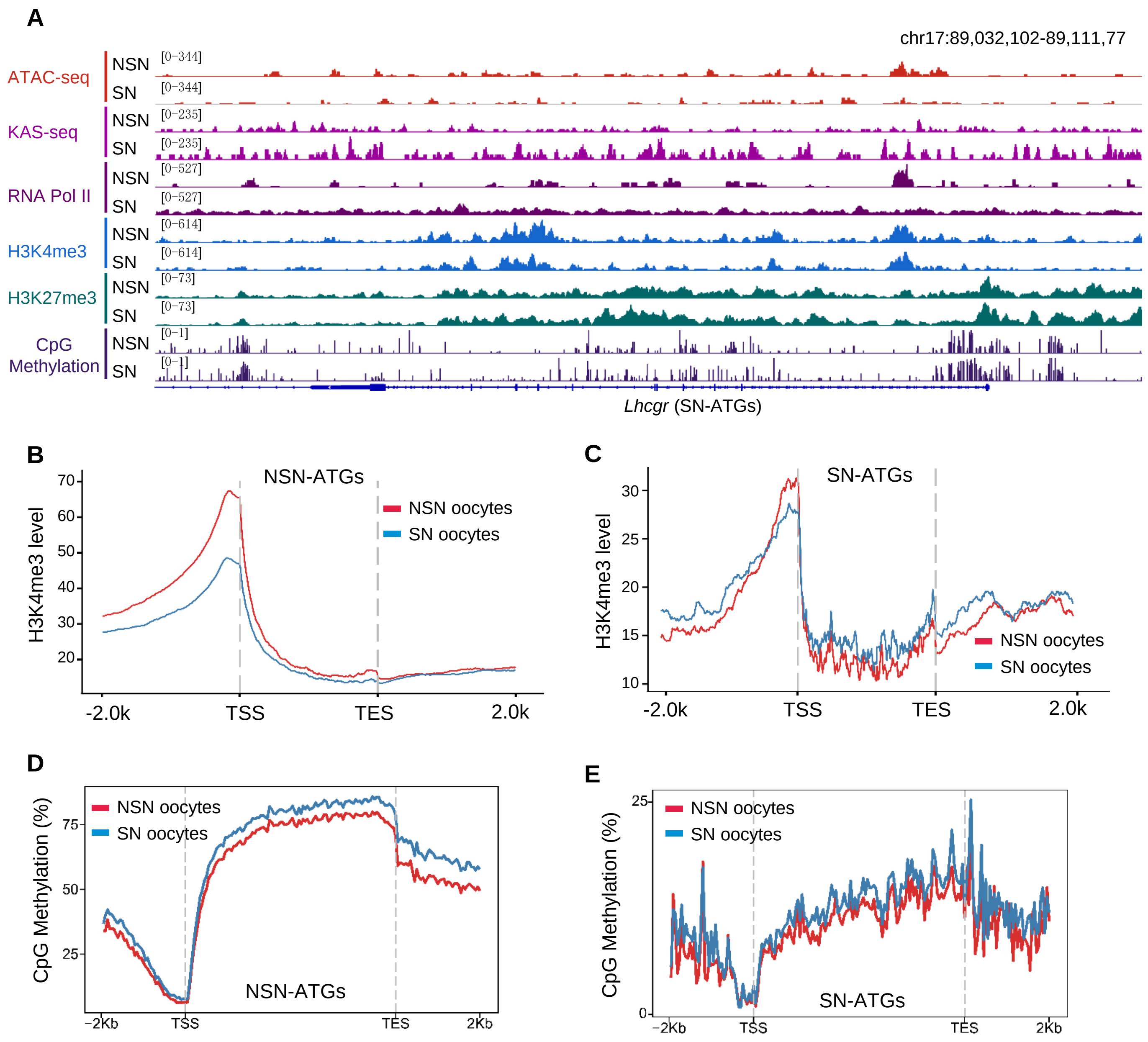

Supplement: Supplementary file 1 — Data S1.Supporting Information. [file CPR-58-e13733-s002.pdf]
